# Supplementary material for: Hyperspectral imaging for dynamic thin film interferometry
Source: Sci Rep. 2020 Jul 9;10:11378. doi: 10.1038/s41598-020-68433-0 (PMC7347853; doi:10.1038/s41598-020-68433-0)
Supplement: Supplementary file 1 — Supplementary material 1 (PDF 13132 kb) [file 41598_2020_68433_MOESM1_ESM.pdf]

SCIENTIFIC REPORTS

Supplementary Information for

Hyperspectral imaging for dynamic thin film interferometry

V. Chandran Suja; J. Sentmanat; G. Hoffman; C. Scales; G. G. Fuller

V. Chandran Suja
Email:vinny@stanford.edu
G. G. Fuller
E-mail: ggf@stanford.edu

This PDF file includes:

Supplementary text
Figs. S1 to S9
Caption for Movie S1
References for SI reference citations

Other supplementary materials for this manuscript include the following:

Movie S1

Supporting Information Text

Robustness against noise

For the numerical experiments quantifying the robustness against noise, the ground truth profile shown in Fig.S1 was converted to interferograms utilizing the relevant colormaps. Subsequently, a Gaussian noise drawn from $\mathcal{N}(0, \sigma^2)$ was superimposed on the interferograms. Here σ is set be three times the standard deviation of the noise encountered at maximum gain in the RGB camera used in this study (Fig.S2). The Signal to Noise Ratio (SNR) ranged from 15 to 20, depending on the filter response function of the camera (Fig.S4). The complete pipeline followed for generating the numerical test cases is available in Fig.S6.

The ideal filters used for the numerical experiments are hat functions arranged contiguously with a width of $200/n$ nm. Here n is the number of channels, and 200 nm is the bandpass window of the global bandpass filter with a cut-on window of $[450, 650]$ nm.

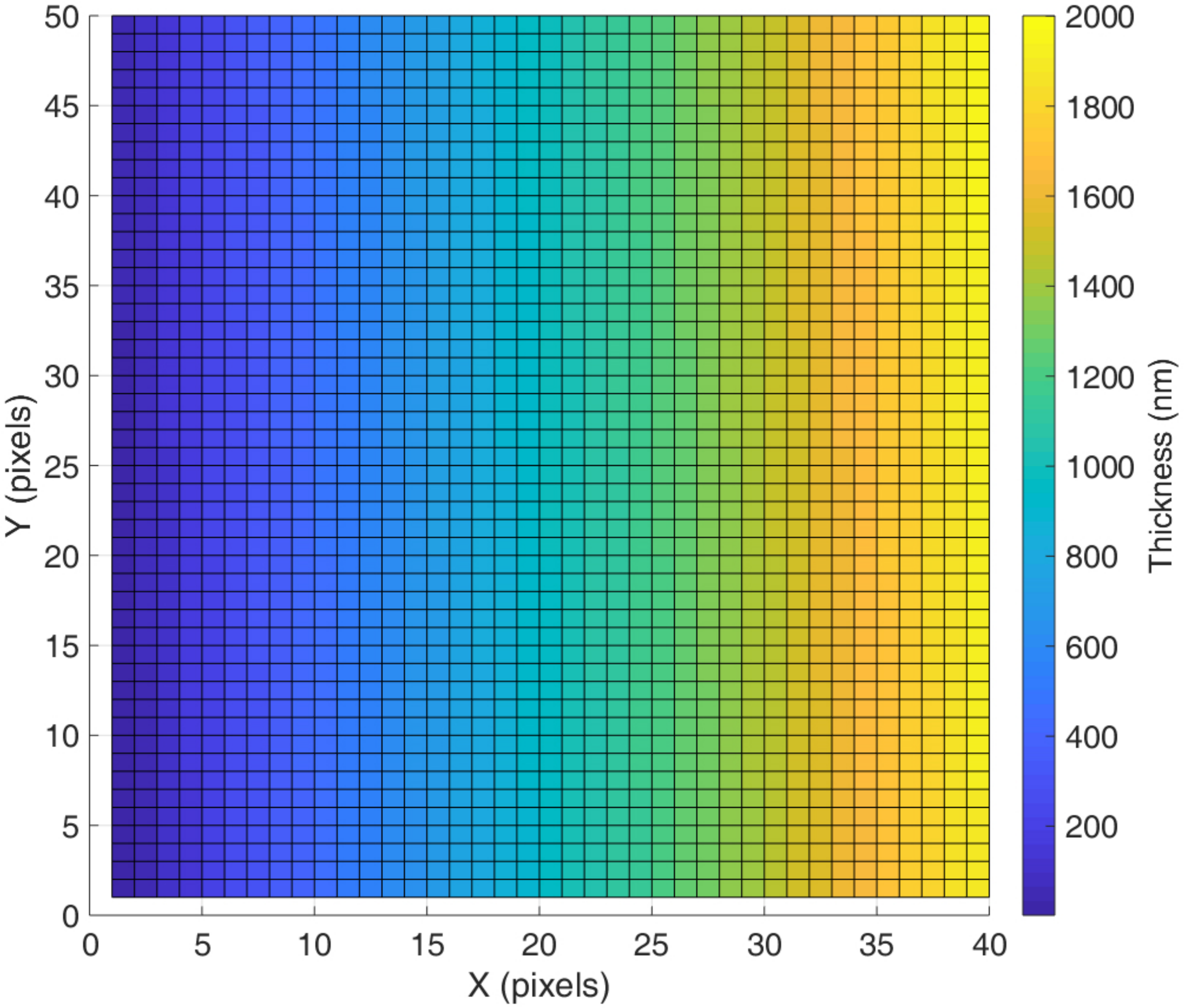

Fig. S1. The heat map of the ground truth profile used for the numerical experiments.

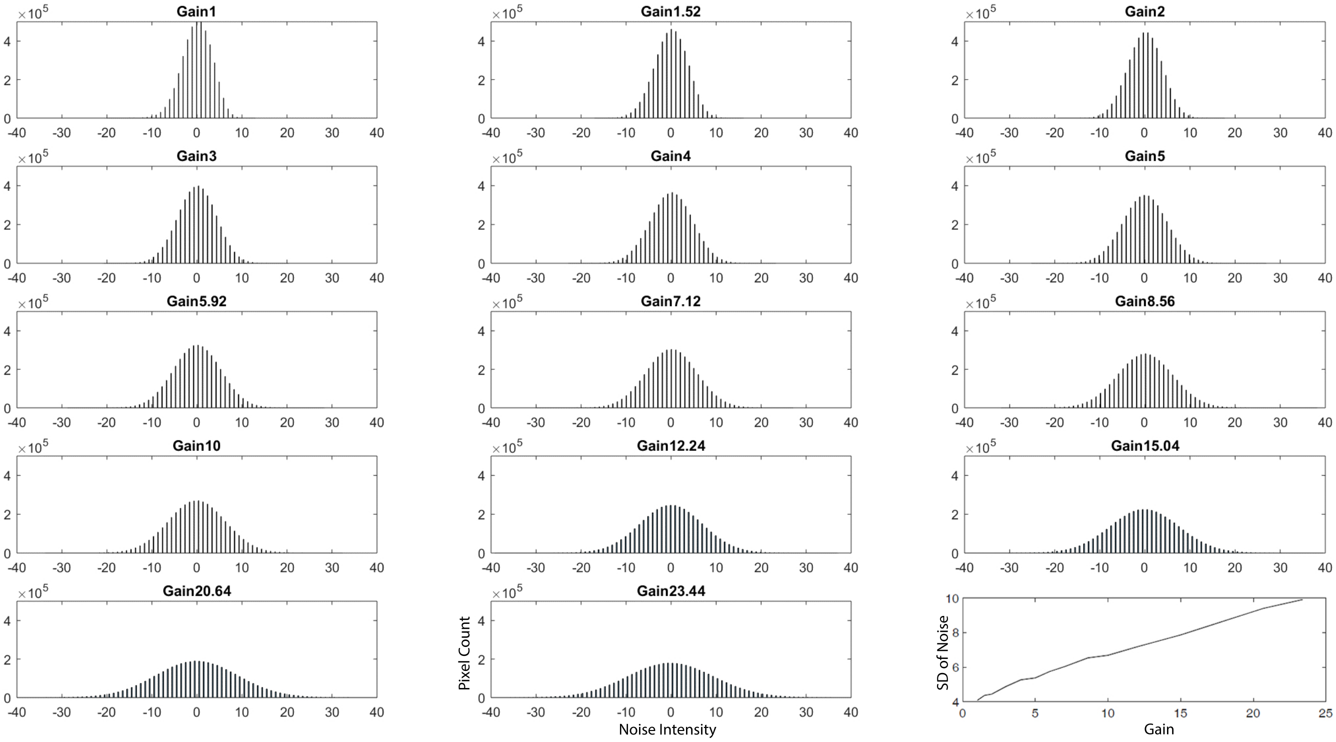

Fig. S2. The measured noise profile in the RGB camera (IDS UI3060P) at different gains

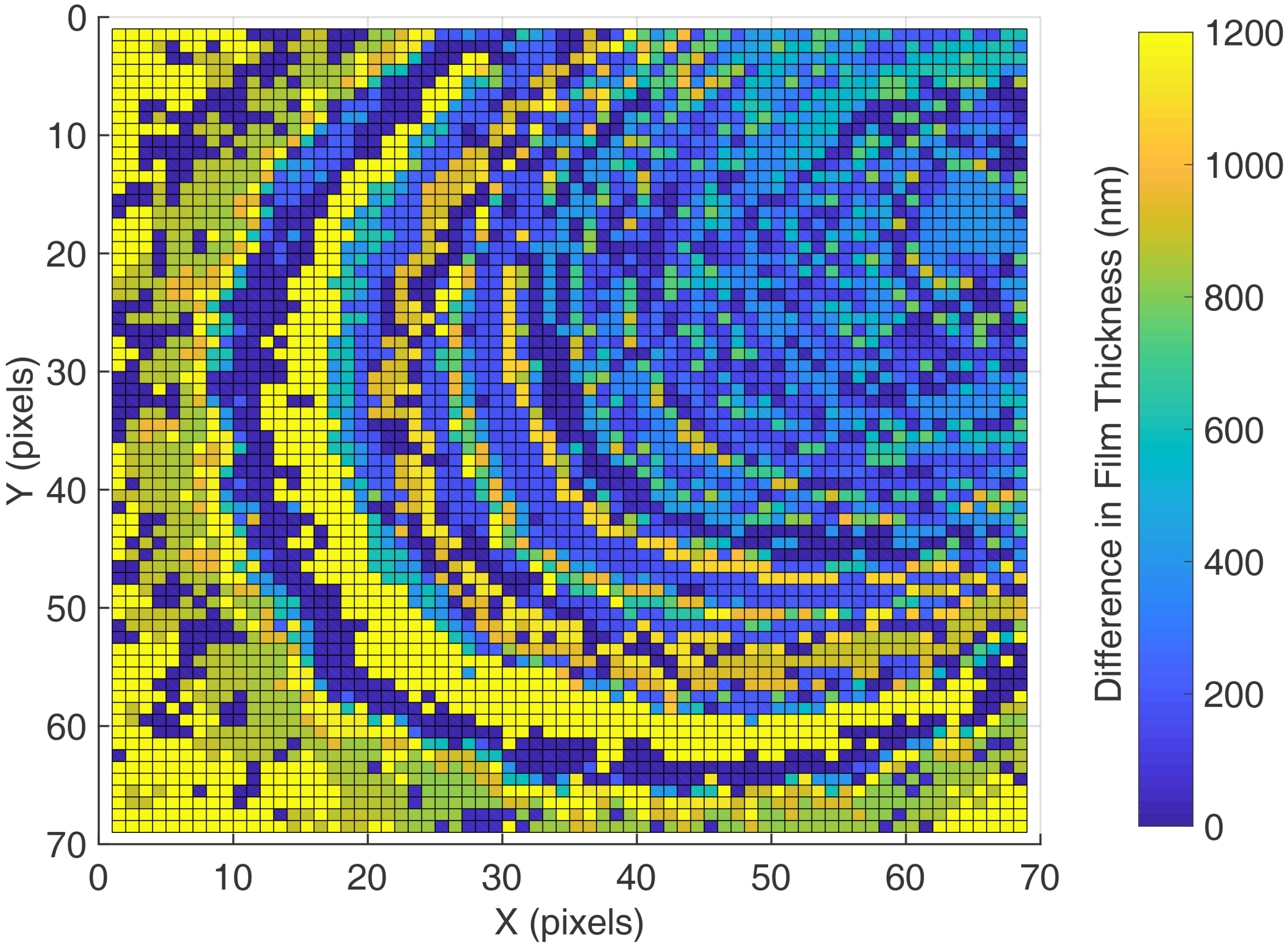

Fig. S3. The pixel wise difference between the manually reconstructed thickness profile and that reconstructed from a 3 channel RGB image. Less than 21% of the pixels differ by $100\ nm$ or less.

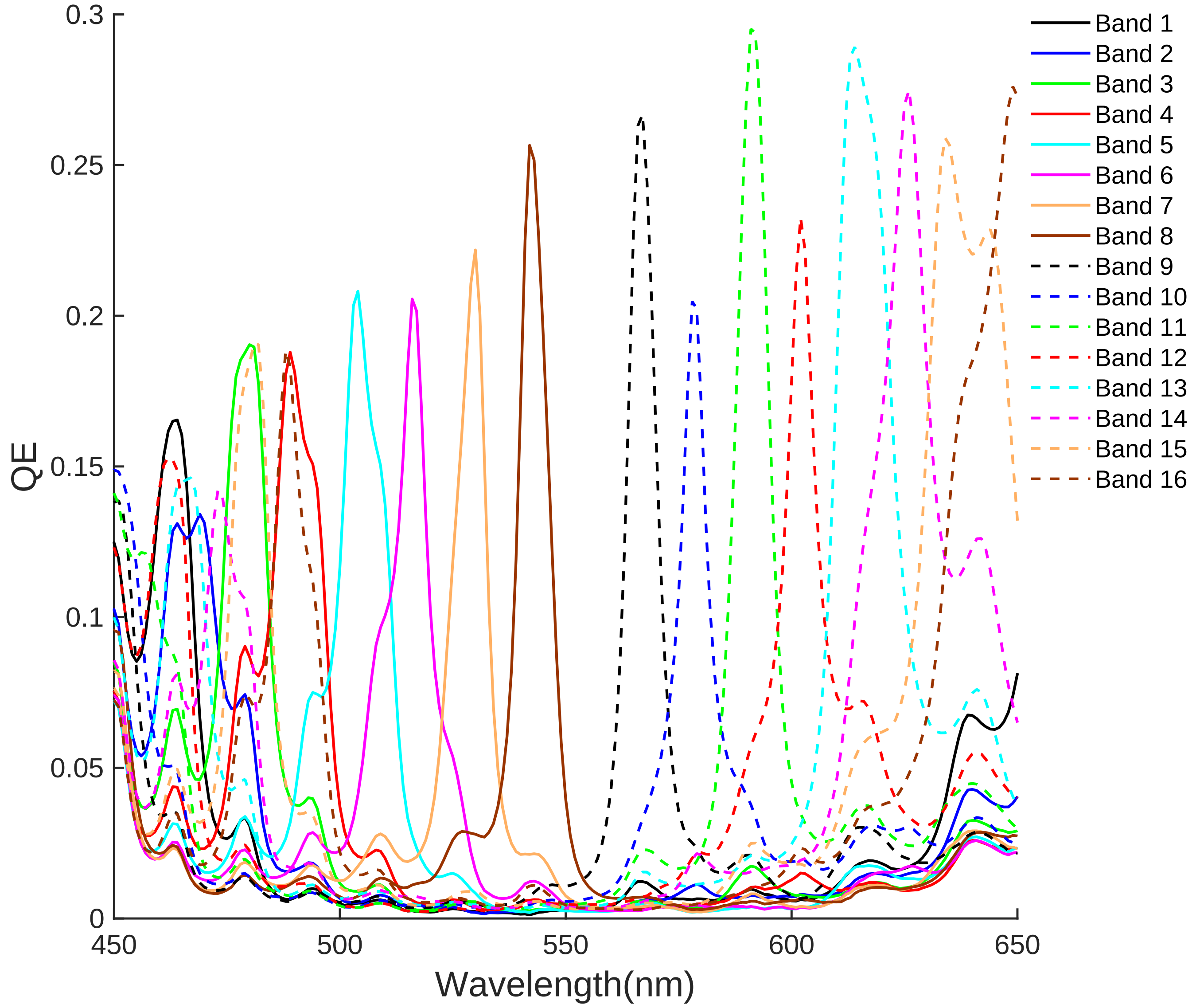

Fig. S4. The filter response function of the Ximea MQ022HG-IM-SM4X4-VIS camera used in the study. The bands are sorted in the ascending order of their peak wavelength. These filters, also called as real filters, are physical Fabry-Perot filters that reside on the camera sensor board.

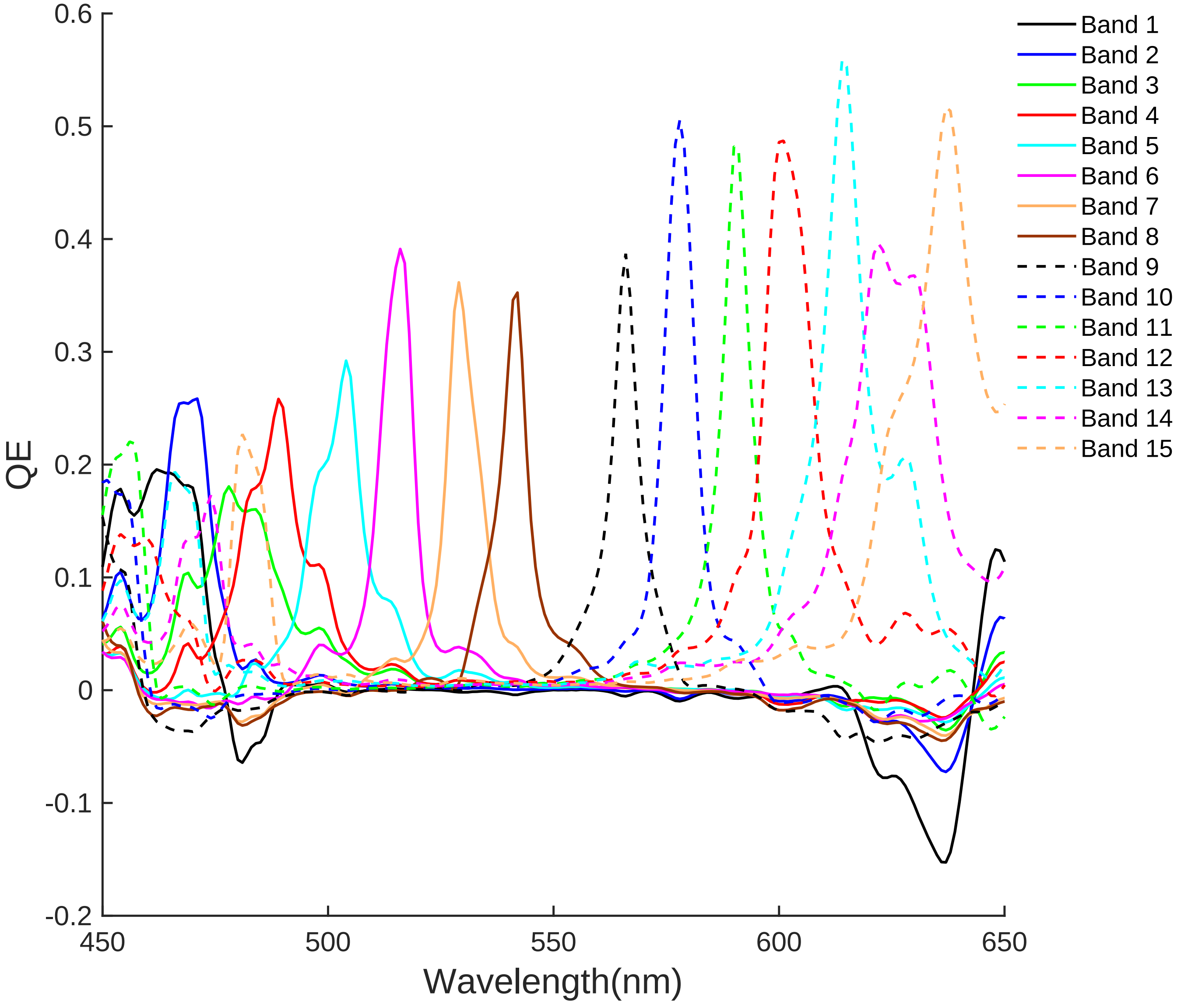

Fig. S5. The virtual filter response function of the Ximea MQ022HG-IM-SM4X4-VIS camera used in the study obtained by multiplying the filter response of the real filters with a spectral correction matrix. The bands are sorted in the ascending order of their peak wavelength. The virtual filter responses are obtained by composing the real filter responses (Fig.S4) with the corresponding correction matrices obtained through spectral calibration. Virtual filters are thus ideal filters that would have generated the spectrally corrected hyperspectral cube.

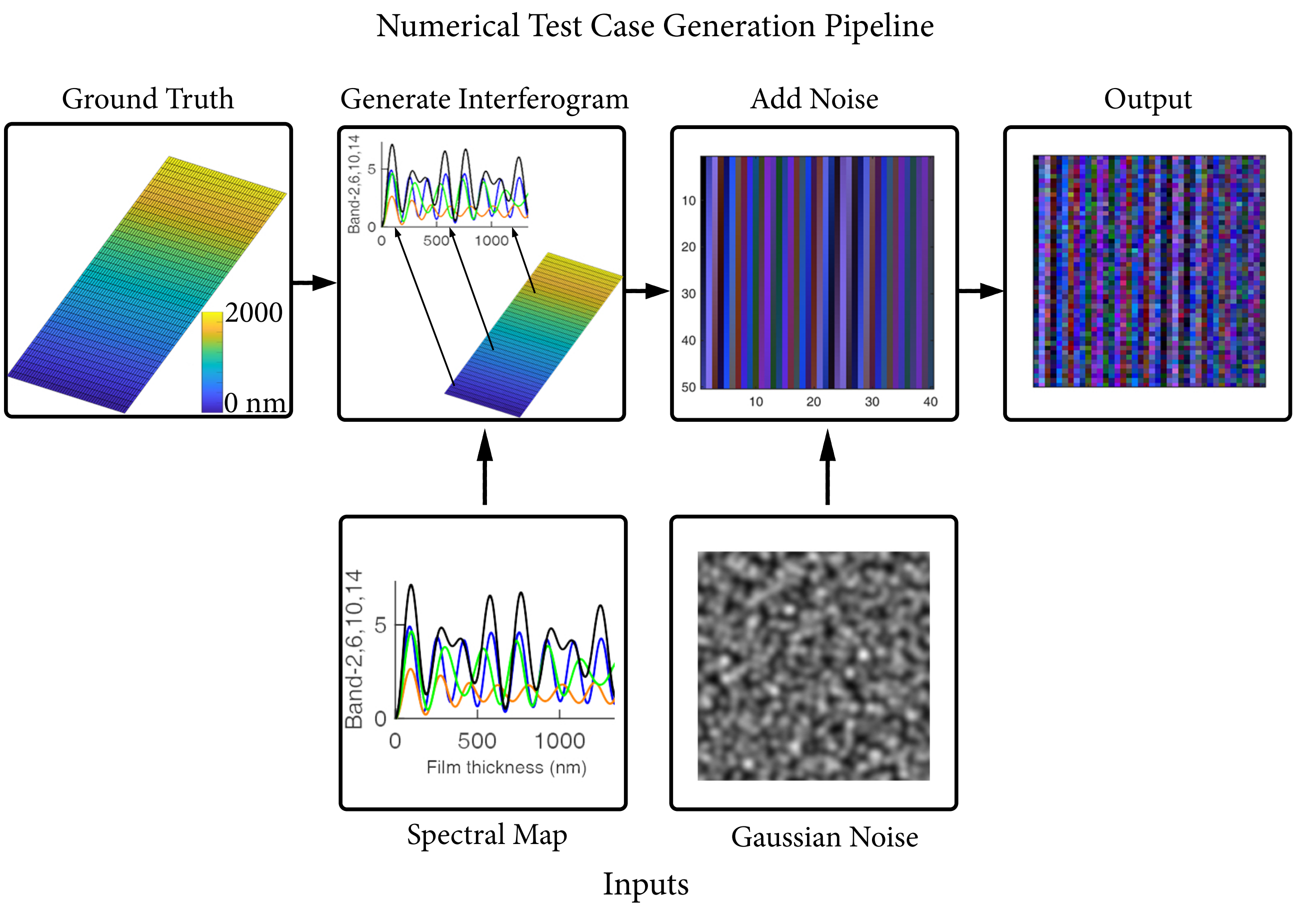

Fig. S6. The pipeline followed for generating the numerical test cases.

Refractive Index Error Analysis

For obtaining the film thickness, the refractive indices of the three media involved are a necessary input. In Fig. S7, we show utilizing numerical experiments, the error in thickness estimation for different magnitudes of error in the estimation of the refractive indices n_1 and n_2. There are three important observations that stand out. Firstly, the error in estimating the refractive index of the media whose thickness is being measured (n_2), influences the accuracy of the measured thickness more than the errors in the refractive index of the bounding media. Secondly, the error in thickness measurement due to an error in n_2 scales with the true thickness. Thirdly, the relative error (absolute error over true thickness) in thickness measurement is proportional to the error in n_2. All these observations are not surprising if we recall that,

$$\frac{I(\lambda, d)}{I_0(\lambda)} = R_1 + R_2(1-R_1)^2 + 2\sqrt{R_1 R_2 (1-R_1)^2}\cos\left(\frac{4\pi n_2 d}{\lambda} + \pi\mathbb{1}(n_2 > n_1) + \pi\mathbb{1}(n_3 > n_2)\right).$$

Clearly,

$$\frac{\partial}{\partial n_2}\left(\frac{I(\lambda, d)}{I_0(\lambda)}\right) \propto \frac{d}{\lambda},$$

while, $\frac{\partial}{\partial n_1}\left(\frac{I(\lambda,d)}{I_0(\lambda)}\right)$ and $\frac{\partial}{\partial n_3}\left(\frac{I(\lambda,d)}{I_0(\lambda)}\right)$ are independent of d, the thickness of the film.

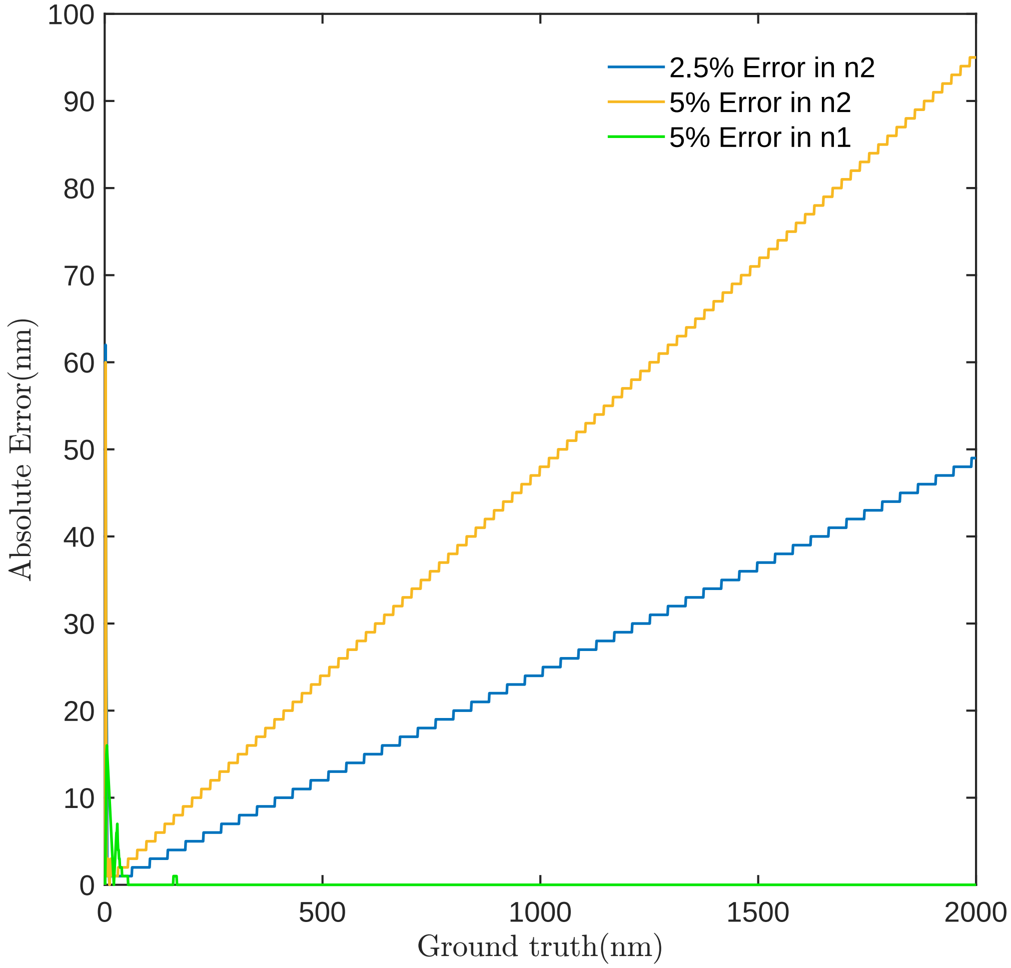

Fig. S7. The results from numerical experiments showing the influence of the error in the refractive index on the accuracy of the calculated thickness. For these numerical experiments, similar to the other numerical experiments reported in the manuscript, a ramp profile (Fig.S1) was constructed and the corresponding interferogram was determined as detailed in Fig.S6. Subsequently, the thickness corresponding to the interferogram was reconstructed with different magnitudes of error imposed on the refractive index.

Statistics of reconstruction accuracy

Fig.S8 shows a histogram categorizing the analyzed pixels as a function of the absolute error. These statics were obtained from a total of ~ 15000 pixels spread across three independent measurements. Prior to optimization, about 84.5% of the pixels are reconstructed with an error of less than 50 nm and 84.6% of the pixels are classified with an error of less than 100 nm. After optimization, about 78.6% of the pixels are reconstructed with an error of less than 50 nm and 95.9% of the pixels are classified with an error of less than 100 nm.

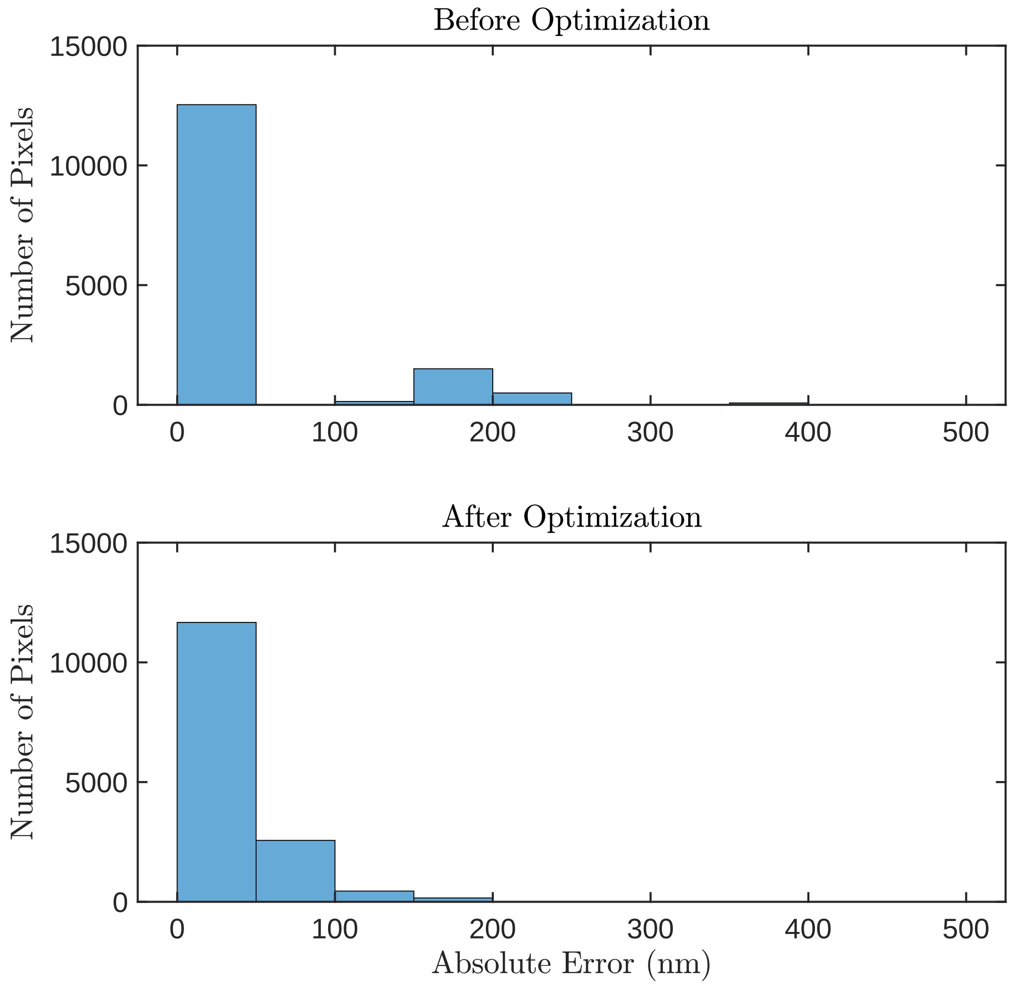

Fig. S8. Histogram categorizing the analyzed pixels as a function of the absolute error.

Position of the camera and Normal Incidence

The position of the camera constrains the angle of incidence of the reflected rays perceived by the camera. Due to the vertical positioning of the lens and the camera in the reported setup, the reflected light rays perceived by the camera are geometrically constrained to those that have a normal or close to a normal angle of incidence ($< 15^o$). The slightly diffuse lighting ensures that a larger portion of spherical liquid films can be imaged, such as in the case of interferograms reported in Fig. 3, which were captured over bubbles in silicone oils. The error in thickness estimation due to assuming a normal incidence is small for the current experimental setup, and the relative error does not exceed 5% (Fig.S9). Further discussion on the influence of curvature on the thickness calculations can be seen in Frostad et al. (1).

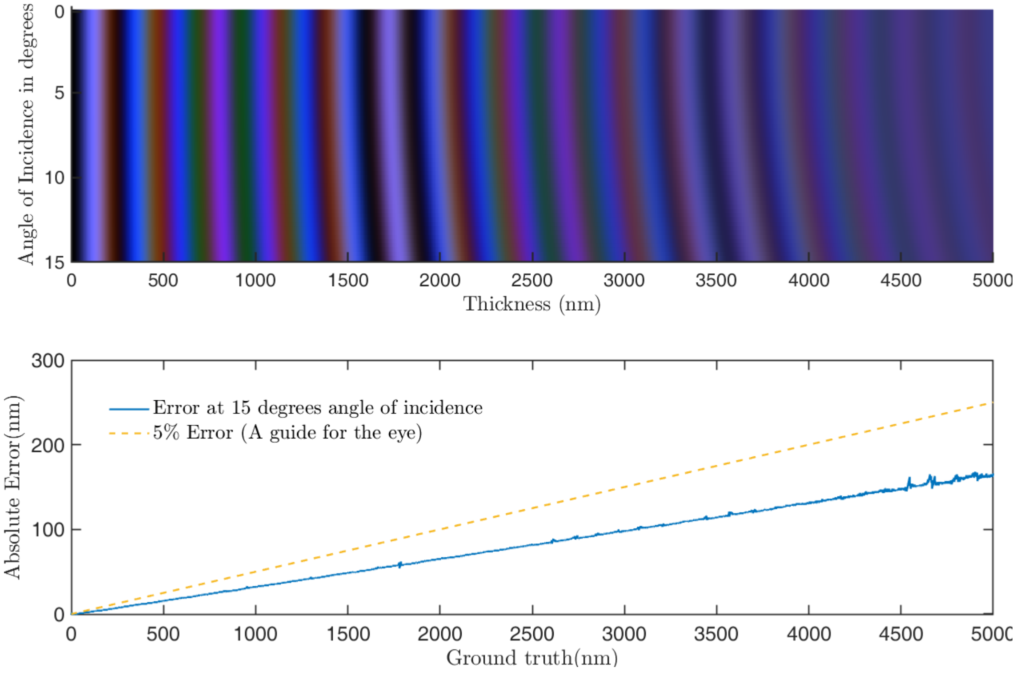

Fig. S9. ***Top:*** A colormap calculated for the IDS UI 3060CP RGB illustrating the influence of the angle of incidence on the obtained interferograms. ***Bottom:*** Absolute error in thickness estimation when assuming normal incidence for reflected light rays having an angle of incidence of 15°. Even at 15°, the maximum expected from geometrical calculations based on the angular extend of the interferogram in Fig. 3 and the corresponding bubble radius, the error is much less than 5%. Note that this error can be significant at higher angles.

Ideal light source and Ideal filters

The ideal light source is a true broadband light source that contains a equal light intensity at all wavelengths in the measurements range. Natural sunlight is very close to an ideal light source in the measurement range. On the other hand, typical LED light sources have very weak light intensity usually near the green wavelength region, resulting in a poor signal to noise ratio for hyperspectral bands in that wavelength range. Having an ideal light source mitigates this issue by improving the signal to noise ratio for all the hyperspectral bands in the camera.

The ideal filter is one which has its spectral responses given by hat functions. In more common terms, these filters have a high 'Q factor'. Unlike a real filter array (see Fig.S4), there is little overlap of the spectral responses for ideal filters. Hence, there is minimal cross talk and every band samples a unique portion of the spectrum, leading to an improved accuracy.

Movie S1. A side by side comparison of a time sequence of interferograms (left) and the automatically reconstructed film thickness profile (right). The video was captured at 10 frames per second.

References

1. Frostad, J. M., Tammaro, D., Santollani, L., de Araujo, S. B. & Fuller, G. G. Dynamic fluid-film interferometry as a predictor of bulk foam properties. *Soft matter* **12**, 9266–9279 (2016).
